# Supplementary material for: Immunosuppressive Activities of Novel PLA2 Inhibitors from Xenorhabdus hominickii, an Entomopathogenic Bacterium
Source: Insects. 2020 Aug 4;11(8):505. doi: 10.3390/insects11080505 (PMC7469199; doi:10.3390/insects11080505)
Supplement: Supplementary file 1 [file insects-11-00505-s001.pdf]

Supplementary data

Table S1. Statistical parameters to estimate median toxicities presented in Table 1

Table S2. Statistical parameters to estimate median toxicities presented in Table 2

Table S3. Statistical parameters to estimate median toxicities presented in Table 3

Figure S1. HPLC chromatograms of four different organic extracts of *X. hominickii* ('Xh') culture broth. Hexane ('HEX'), ethyl acetate ('EAX'), chloroform ('CX'), and butanol ('BX') organic solvents were used to extract metabolites.

Figure S2. Screening 15 subfractions ('F1-F15') of butanol extract against PLA_2 hemocyte PLA_2 activity of *S. exigua*. Each measurement was replicated three times. Different letters above standard deviation bars indicate significant differences among means at Type I error = 0.05 (LSD test).

Figure S3. Subfractions of two potent fractions ('F2 and F6'). (A) HPLC analysis of F2 and F6. (B) Preparatory TLC to isolate compounds in each subfraction. Eluent composed of chloroform, methanol, and acetic acid (7.5:2:0.5, v/v) in the silica plates.

Figure S4. HPLC analysis of eight active subfractions ('F2-1, F2-2, F2-6, F2-8, F6-3, F6-4, F6-8, and F6-9') from butanol extracts of *X. hominickii* culture broth.

Figure S5. GC-MS analysis of eight active subfractions ('F2-1, F2-2, F2-6, F2-8, F6-3, F6-4, F6-8, and F6-9') from *X. hominickii* culture broth. Predicted compounds are indicated by

arrows among GC peaks. These include dioctyl terephthalate (DOTP) from F2-1, 3-ethoxy-4-methoxyphenol (EMP) from F2-2, bis(2-ethylhexyl) phthalate (BEP) from F2-6, 2-ethyl-1-hexanol (EH) from F2-8, docosane (DS) from F6-3, phthalimide (PM) from F6-4, o-cyanobenzoic acid (CBA) from F6-8, dibutylamine (DBA) from F6-9.

Figure S6. Enhanced insecticidal activities of bacterial pathogens by eight PLA_2 inhibitors: dioctyl terephthalate (DOTP), 3-ethoxy-4-methoxyphenol (EMP), bis(2-ethylhexyl) phthalate (BEP), 2-ethyl-1-hexanol (EH), docosane (DS), phthalimide (PM), o-cyanobenzoic acid (CBA), and dibutylamine (DBA). (A) Enhanced effect of the inhibitors on *X. hominickii* ('Xh') pathogenicity. L5 larvae of *S. exigua* were hemocoelically injected with low dose (10^2 cfu/larva) of Xh or along with the PLA_2 inhibitors (2 μg/larva). (B) Enhanced effect of the inhibitors on *Bacillus thuringiensis* ('Bt') pathogenicity. A small piece (2 cm^2) of cabbage leaf was dipped in Bt (500 ppm) or a mixture with PLA_2 inhibitor (1,000 ppm). The treated leaves were provided to test *S. exigua* larvae. Each treatment was replicated three times with 10 insects per replication. Different letters above standard deviation bars represent significant differences among means at Type I error = 0.05 (LSD test). Insecticidal activity was assessed at 4 days after treatment (DAT).

Table S1. Statistical parameters to estimate median toxicities presented in Table 1

Inhibitors	PLA_2	t	IC_{50} (95% CI)	Slope	df	*P*	SE
BEP	Total	0.8864	0.17 (0.09-0.32)	0.241	4	0.4255	0.014
	$sPLA_2$	1.1301	0.07 (0.04-0.13)	0.2352	4	0.3216	0.013
	$cPLA_2$	1.3396	0.09 (0.05-0.16)	0.2750	4	0.2514	0.11
CBA	Total	0.6183	0.86 (0.46-1.56)	0.244	4	0.5699	0.02
	$sPLA_2$	0.4186	1.11 (0.58-2.21)	0.197	4	0.7105	0.11
	$cPLA_2$	0.7448	0.38 (0.20-0.71)	0.2651	4	0.4978	0.034
DBA	Total	1.1182	0.18 (0.95-0.35)	0.249	4	0.3261	0.024
	$sPLA_2$	2.1514	0.11 (0.06-0.21)	0.2777	4	0.0978	00.024
	$cPLA_2$	1.586	0.19 (0.11-0.35)	0.2788	4	0.1878	00.022
DOTP	Total	2.0127	0.16 (0.91-0.29)	0.2765	4	0.1144	0.0064
	$sPLA_2$	0.3731	0.56 (0.30-1.1)	0.1878	4	0.728	0.054
	$cPLA_2$	1.1909	0.06 (0.04-0.11)	0.2584	4	0.2995	0.051
DS	Total	0.9750	0.81 (0.43-1.55)	0.2675	4	0.3848	0.044
	$sPLA_2$	0.1846	1.22 (0.62-2.21)	0.1722	4	0.8625	0.154
	$cPLA_2$	1.0165	0.32 (0.15-0.61)	0.2947	4	0.3669	0.024
EH	Total	0.6383	0.14 (0.08-0.29)	0.2441	4	0.5580	0.014
	$sPLA_2$	1.8931	0.05 (0.3-0.10)	0.238	4	0.1313	0.0044
	$cPLA_2$	3.0785	0.11 (0.06-0.20)	0.2742	4	0.0369	0.014
EMP	Total	1.4462	0.04 (0.02-0.07)	0.2684	4	0.2217	0.023
	$sPLA_2$	2.0511	0.03 (0.01-0.05)	0.2763	4	0.1096	0.0054
	$cPLA_2$	0.9557	0.05 (0.03-0.10)	0.2524	4	0.3934	0.0041
PM	Total	1.5955	0.05 (0.03-0.13)	0.2678	4	0.1858	0.0074
	$sPLA_2$	2.1430	0.17 (0.10-0.32)	0.2520	4	0.0988	0.014
	$cPLA_2$	1.052	0.04 (0.02-0.07)	0.2632	4	0.3520	0.0034

Table S2. Statistical parameters to estimate median toxicities presented in Table 2

Inhibitors	Type	t	IC_{50} (95% CI)	Slope	df	*P*	SE
BEP	Nodulation	2.517	1.0 (0.54-1.91)	0.3539	4	0.0656	0.11
	Phagocytosis	5.980	1.50 (0.81-2.89)	0.3171	4	0.0039	0.22
CBA	Nodulation	5.025	2.50 (1.35-5.1)	0.4621	4	0.0074	0.22
	Phagocytosis	2.4629	32.50 (17.10-63.0)	0.378	4	0.0695	2.35
DBA	Nodulation	2.681	0.71 (0.38-1.38)	0.4139	4	0.055	0.05
	Phagocytosis	4.15	5.0 (2.8-9.4)	0.3112	4	0.0143	0.58
DOTP	Nodulation	1.5104	1.10 (0.61-2.12)	0.2812	4	0.205	0.07
	Phagocytosis	1.5069	8.70 (4.6-16.4)	0.2613	4	0.2063	0.89
DS	Nodulation	2.0476	1.11 (0.54-2.21)	0.336	4	0.111	0.09
	Phagocytosis	1.5485	8.40 (4.6-9.1)	0.3287	4	0.1964	0.68
EH	Nodulation	1.723	0.40 (0.24-0.76)	0.3537	4	0.160	0.03
	Phagocytosis	2.480	0.50 (0.28-0.96)	0.3479	4	0.0678	0.03
EMP	Nodulation	3.188	0.20 (0.11-0.39)	0.3980	4	0.0332	0.02
	Phagocytosis	4.190	0.20 (0.12-0.37)	0.3201	4	0.0138	0.03
PM	Nodulation	2.704	0.20 (0.11-0.39)	0.3734	4	0.054	0.02
	Phagocytosis	2.571	0.40 (0.22-0.78)	0.3296	4	0.0619	0.05

Table S3. Statistical parameters to estimate median toxicities presented in Table 3

Inhibitors	Type	χ2	IC_{50} (95% CI)	Slope	df	SE
BEP	Injection	0.870	34.88 (17.94-67.45)	0.310	4	0.603
	Feeding	0.955	2652.72 (1345.2-5268.9)	0.378	4	0.496
CBA	Injection	0.985	54.96 (28.2-106.7)	0.321	4	0.599
	Feeding	0.984	2870.40 (1433.6-5679.2)	0.381	4	0.494
DBA	Injection	0.856	38.77 (19.89-75.87)	0.316	4	0.597
	Feeding	0.988	3108.8 (1556.5-6210.9)	0.385	4	0.492
DOTP	Injection	0.856	38.89 (20.4-76.3)	0.315	4	0.597
	Feeding	0.930	3232.9 (1621.5-6458.9)	0.356	4	0.522
DS	Injection	0.836	49.54 (25.3-95.3)	0.298	4	0.629
	Feeding	0.971	3629.21 (1804.6-7243.8)	0.369	4	0.511
EH	Injection	0.878	29.90 (15.8-55.2)	0.324	4	0.582
	Feeding	0.965	2451.52 (1236.9-4850.1)	0.374	4	0.498
EMP	Injection	0.876	18.95 (10.5-33.7)	0.339	4	0.554
	Feeding	1.00	2160.10 (1091.5-4259.8)	0.335	4	0.541
PM	Injection	0.906	22.11 (12.7-42.6)	0.336	4	0.560
	Feeding	0.995	2177.32 (1092.5-4320.4)	0.350	4	0.524

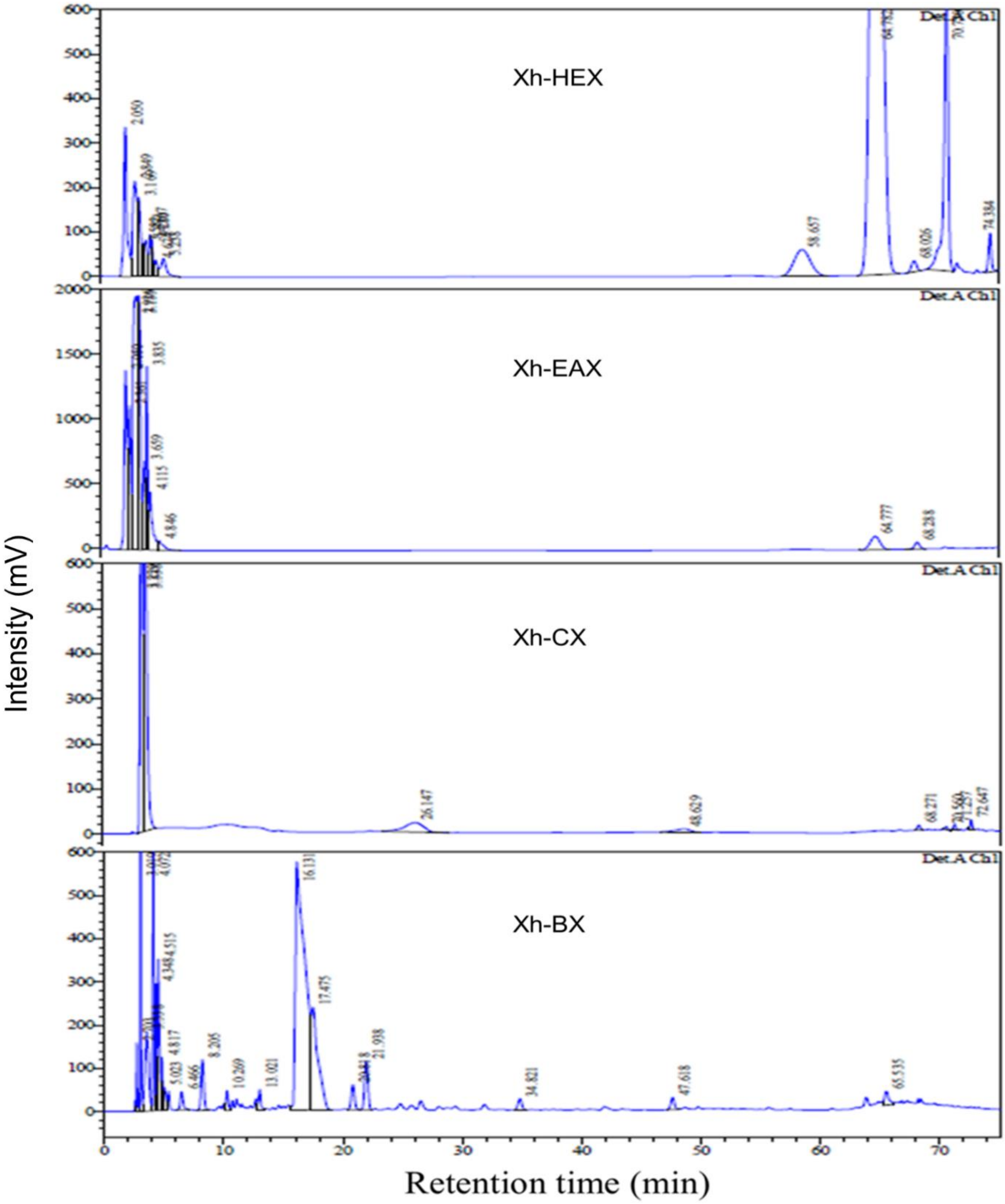
Xh-HEX
Xh-EAX
Xh-CX
Xh-BX
Det.A Ch1
Intensity (mV)
Retention time (min)
58.657
68.026
74.384
64.777
68.288
26.147
48.629
68.271
72.647
16.131
17.475
21.938
8.205
10.269
13.021
34.821
47.618
65.535

Fig. S1

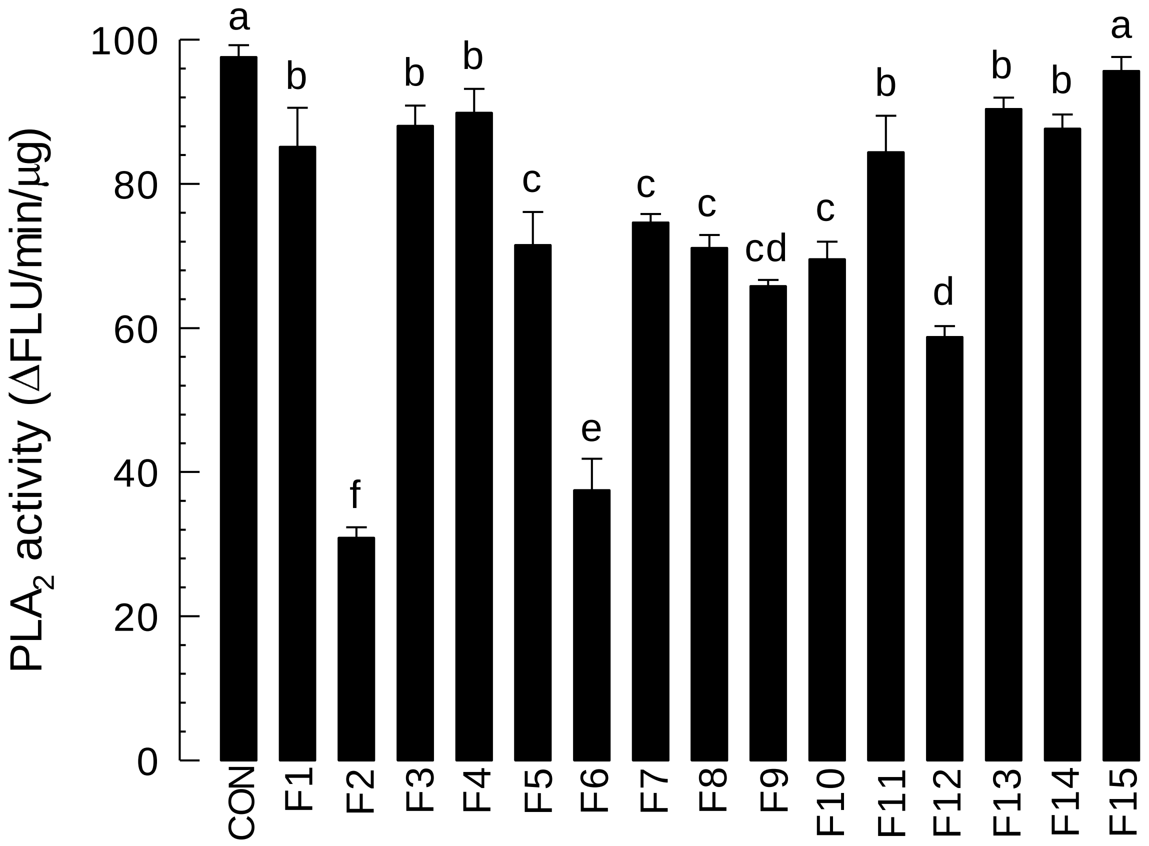

PLA2 activity (ΔFLU/min/μg)
0
20
40
60
80
100
a
b
f
b
b
c
e
c
c
cd
c
b
d
b
b
a
CON
F1
F2
F3
F4
F5
F6
F7
F8
F9
F10
F11
F12
F13
F14
F15

Fig. S2

(A)

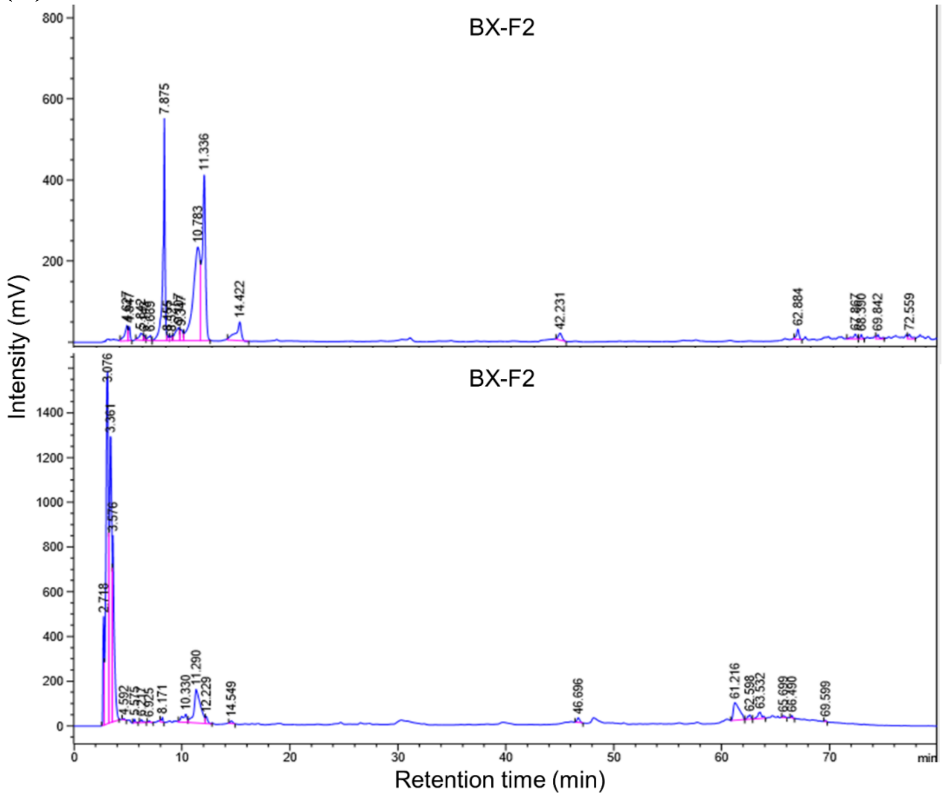
BX-F2
7.875
11.336
10.783
14.422
42.231
62.884
69.842
72.559
BX-F2
3.076
3.361
3.576
2.718
11.290
10.330
12.229
8.171
14.549
46.696
61.216
62.598
63.532
69.599
Intensity (mV)
Retention time (min)
min

(B)

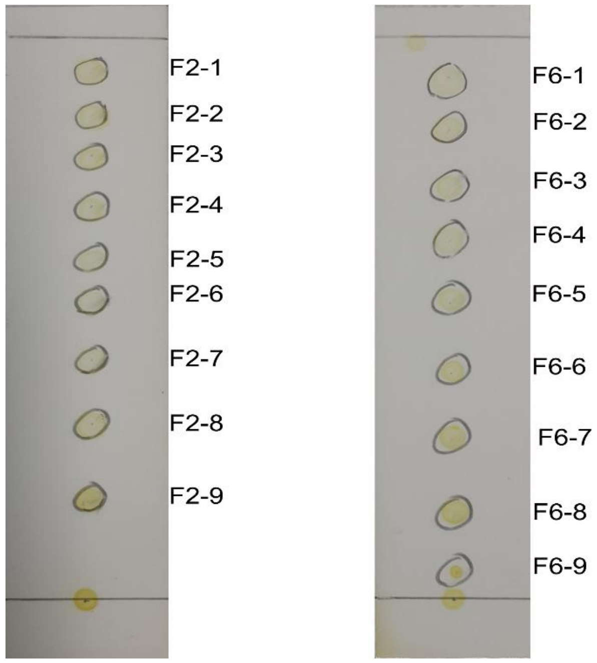
F2-1
F2-2
F2-3
F2-4
F2-5
F2-6
F2-7
F2-8
F2-9
F6-1
F6-2
F6-3
F6-4
F6-5
F6-6
F6-7
F6-8
F6-9

Fig. S3

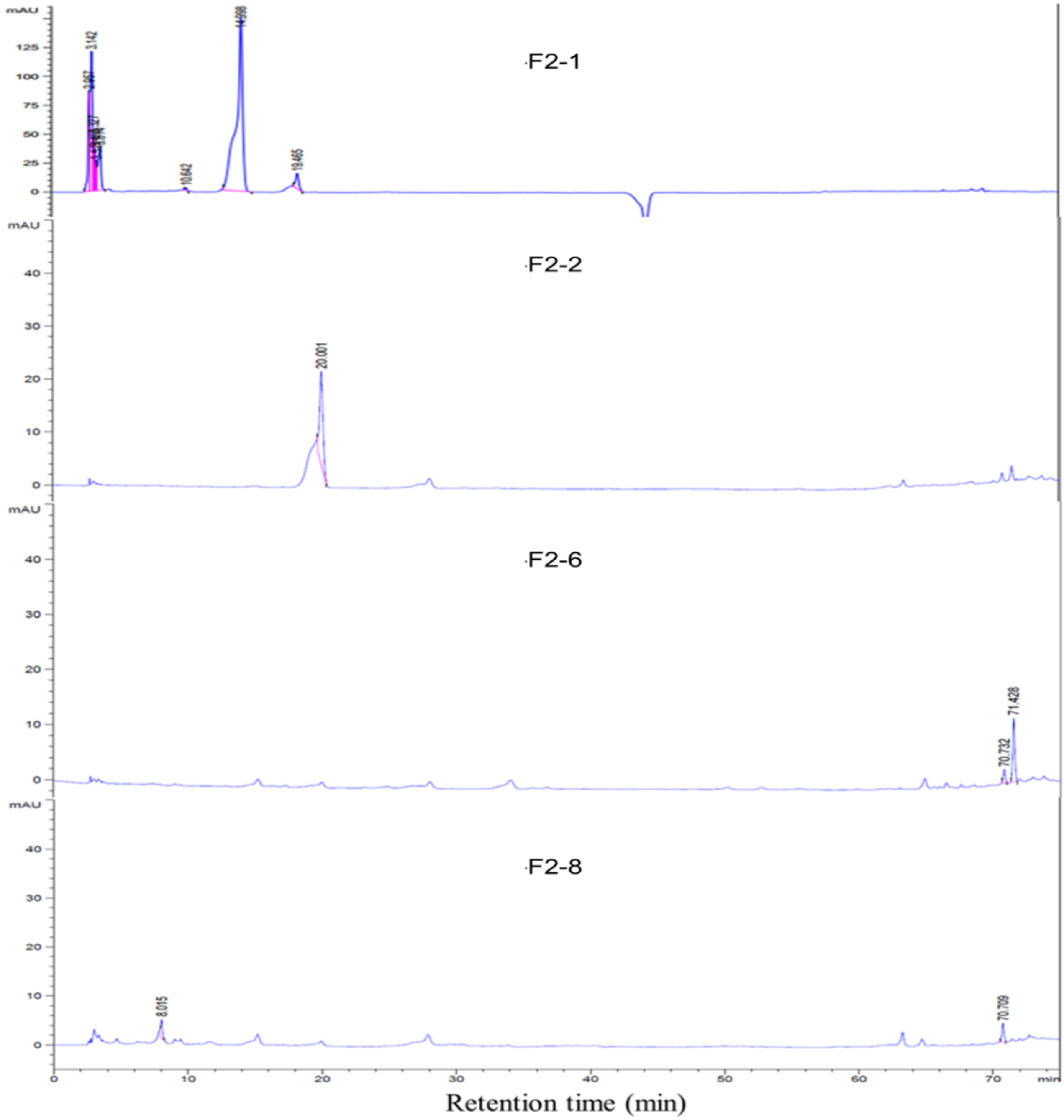
F2-1
F2-2
F2-6
F2-8
mAU
3.142
10.642
18.465
20.001
70.732
71.428
8.015
70.709
min
Retention time (min)

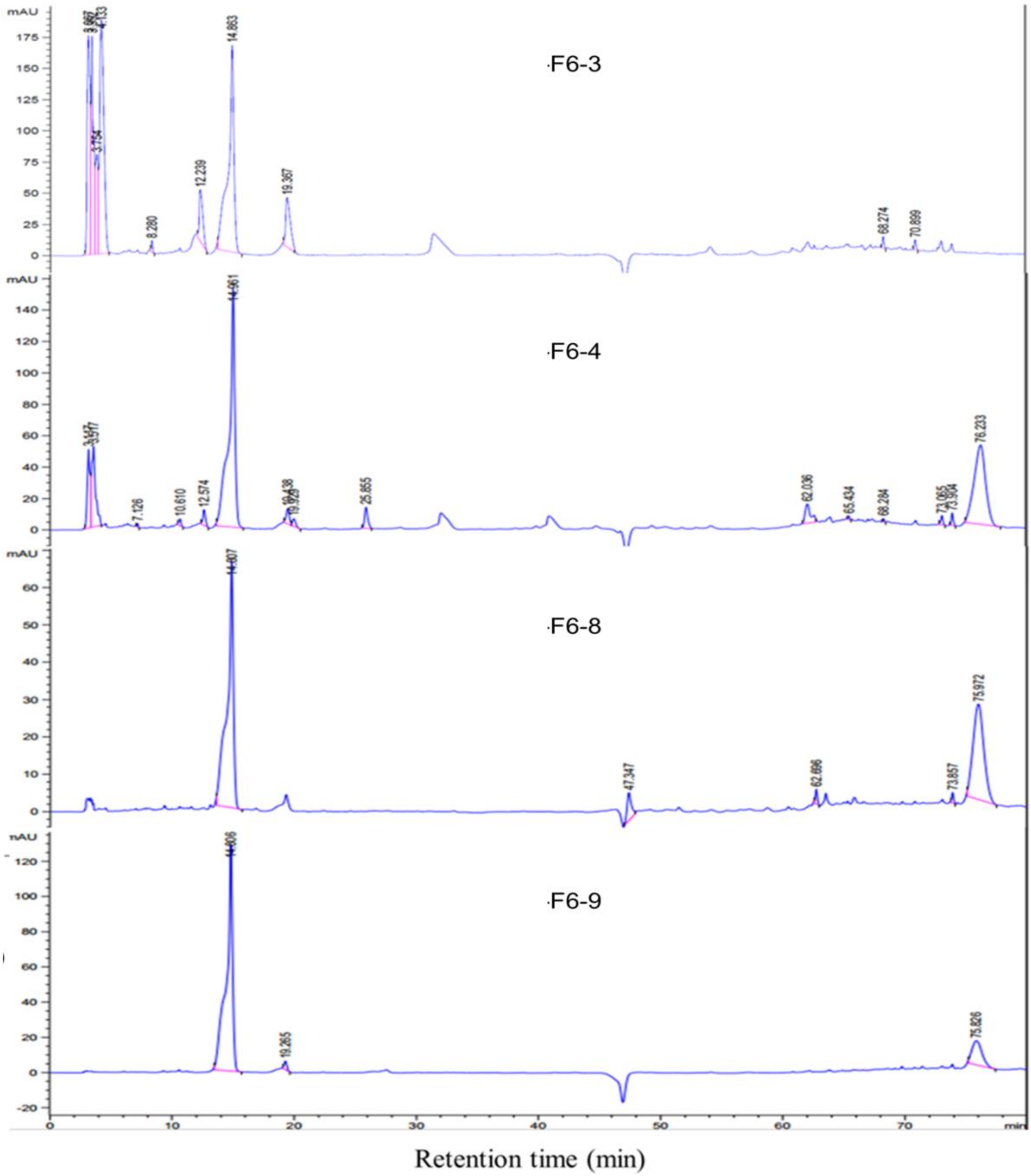

Fig. S4

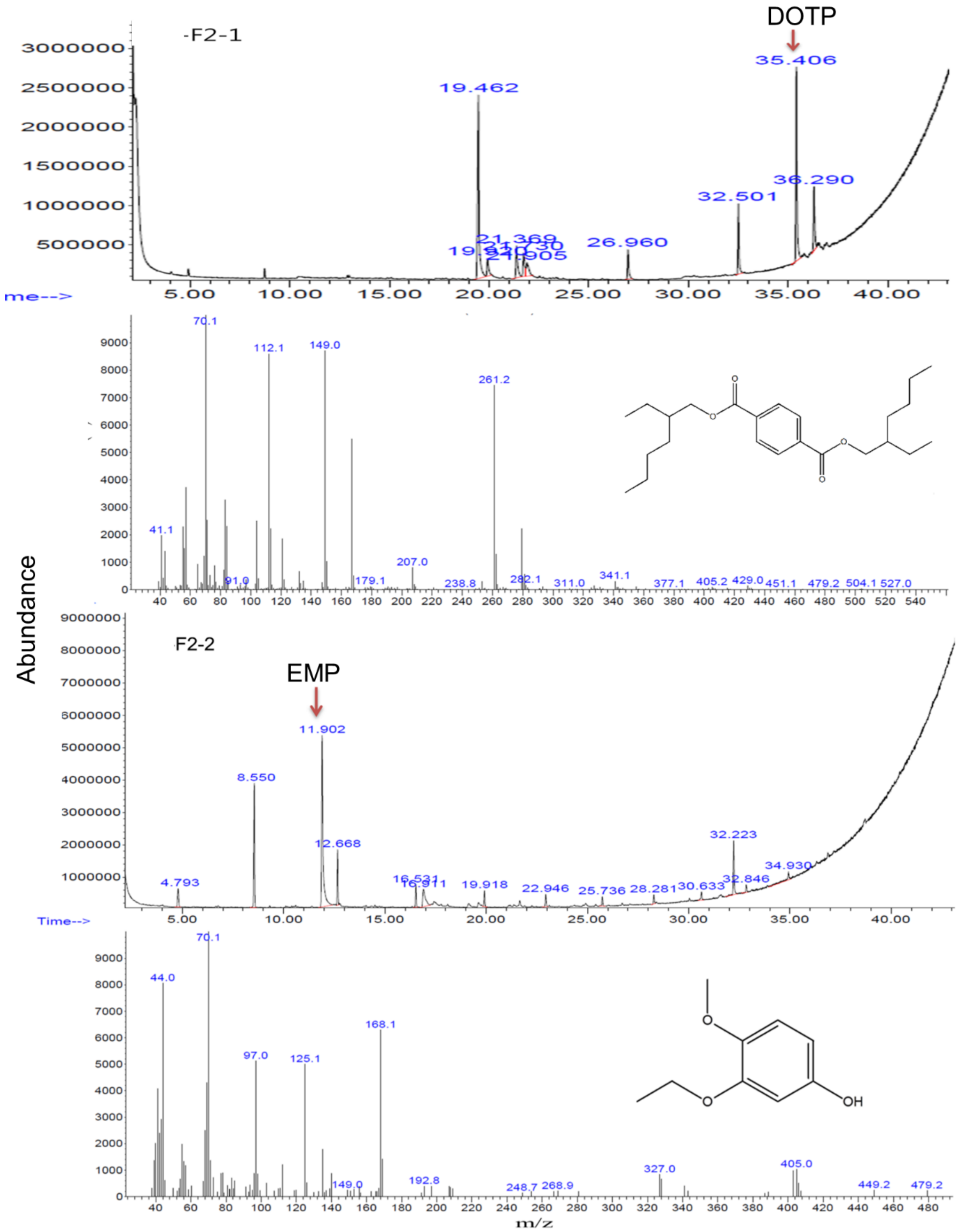

DOTP
-F2-1
19.462
21.369
26.960
32.501
35.406
36.290
me-->
5.00
10.00
15.00
20.00
25.00
30.00
35.00
40.00
70.1
112.1
149.0
261.2
41.1
91.0
179.1
207.0
238.8
282.1
311.0
341.1
377.1
405.2
429.0
451.1
479.2
504.1
527.0
Abundance
F2-2
EMP
11.902
8.550
12.668
4.793
19.918
22.946
25.736
28.281
30.633
32.223
32.846
34.930
Time-->
44.0
70.1
97.0
125.1
168.1
149.0
192.8
248.7
268.9
327.0
405.0
449.2
479.2
OH
m/z

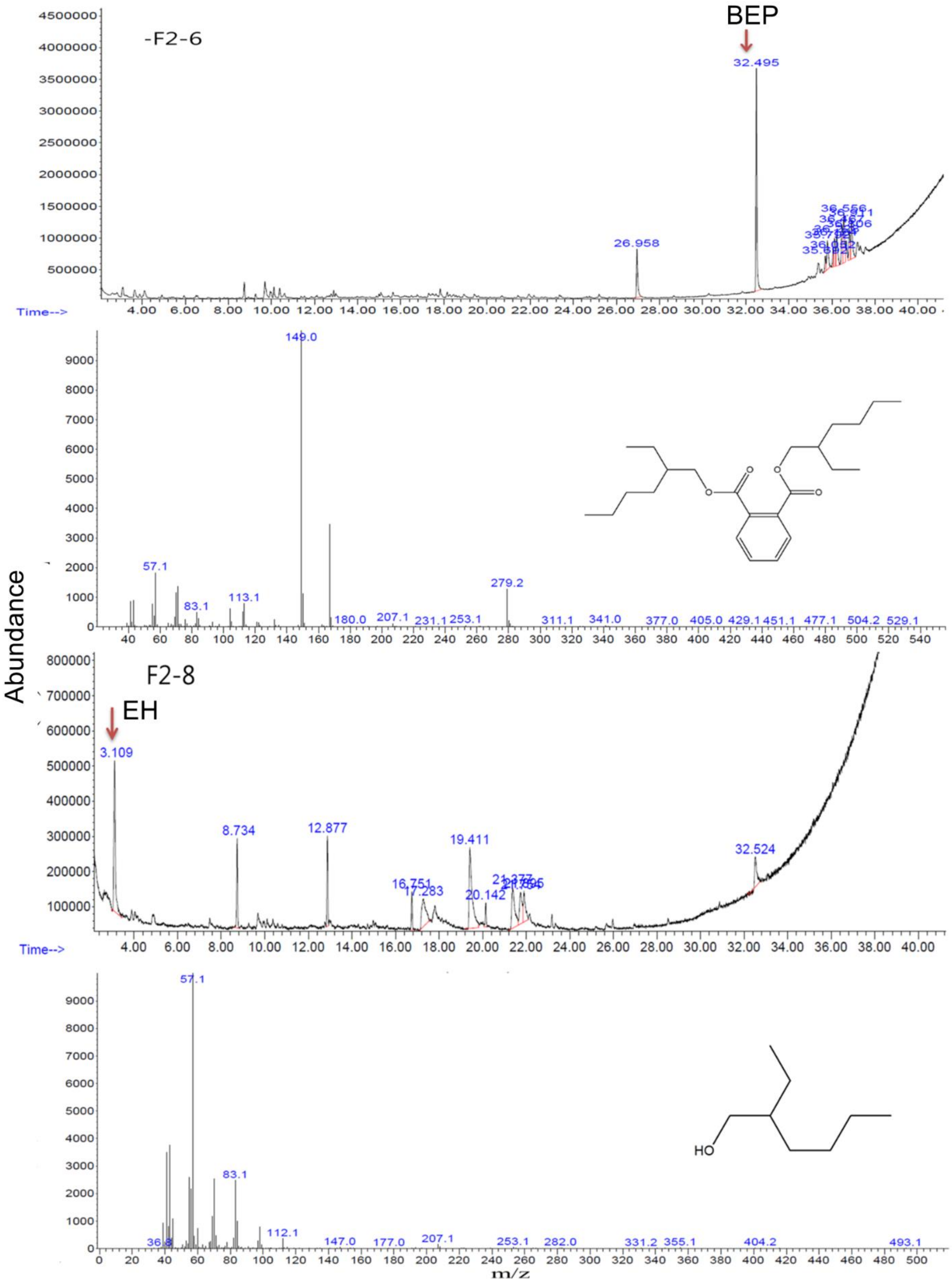

-F2-6
BEP
32.495
26.958
Time-->
149.0
57.1
83.1
113.1
279.2
F2-8
EH
3.109
8.734
12.877
19.411
16.751
17.283
20.142
32.524
57.1
83.1
112.1
HO
Abundance
m/z

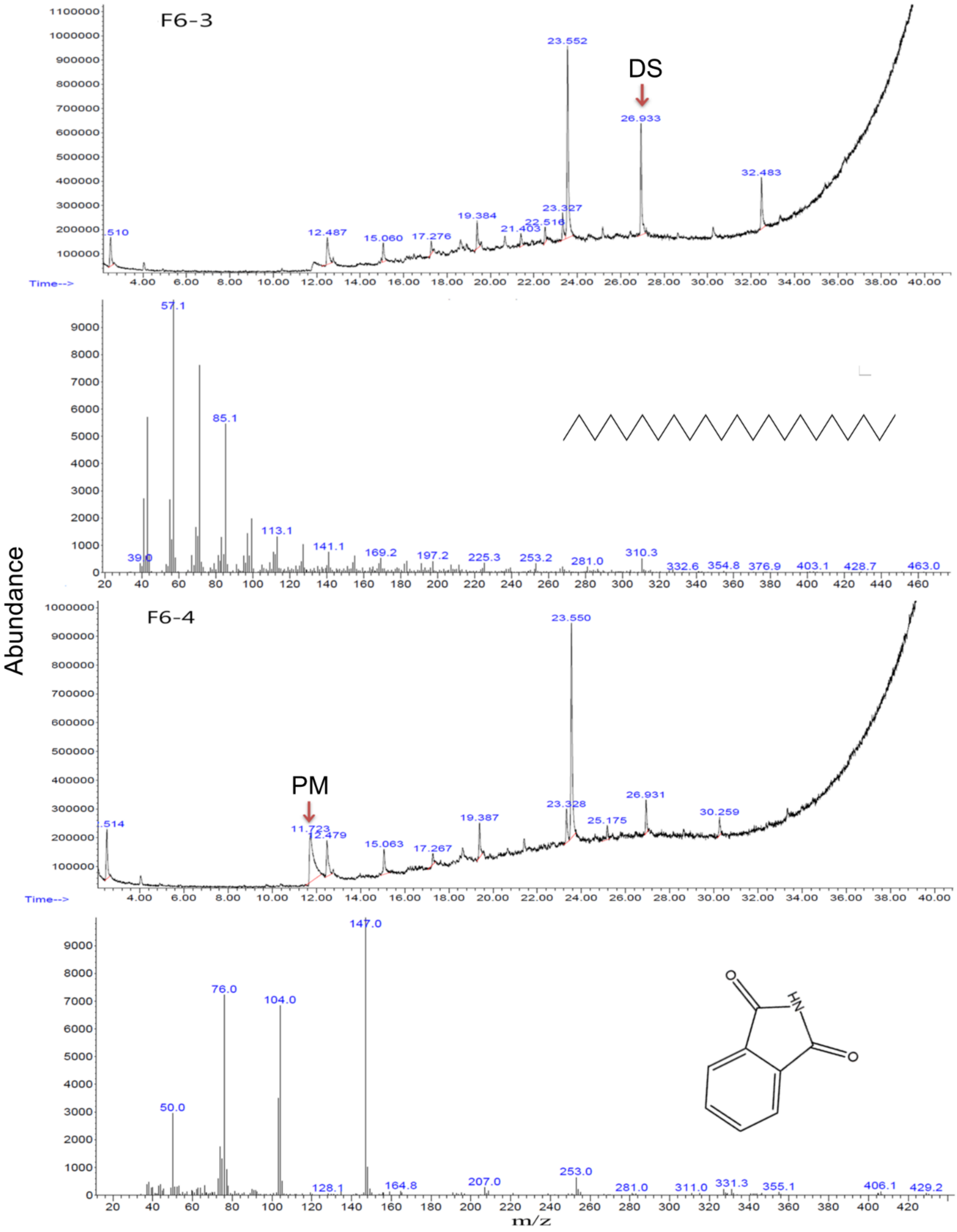

F6-3
DS
23.552
26.933
32.483
19.384
23.327
22.516
21.403
12.487
15.060
17.276
.510
Time-->
57.1
85.1
113.1
141.1
169.2
197.2
225.3
253.2
281.0
310.3
332.6
354.8
376.9
403.1
428.7
463.0
39.0
Abundance
F6-4
PM
23.550
11.723
12.479
15.063
17.267
19.387
23.328
25.175
26.931
30.259
.514
147.0
76.0
104.0
50.0
128.1
164.8
207.0
253.0
281.0
311.0
331.3
355.1
406.1
429.2
m/z
O
HN
O

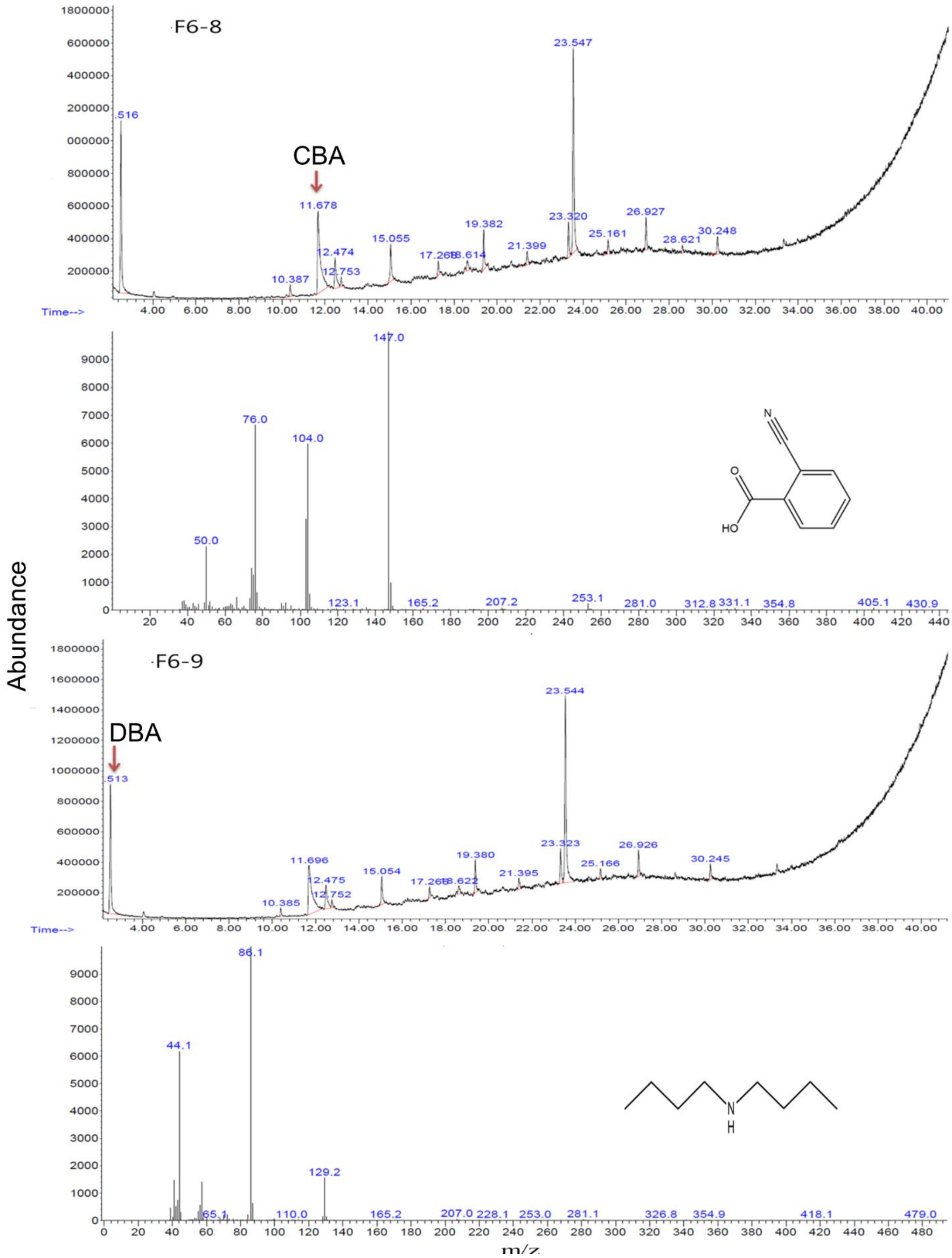

Fig. S5

(A)

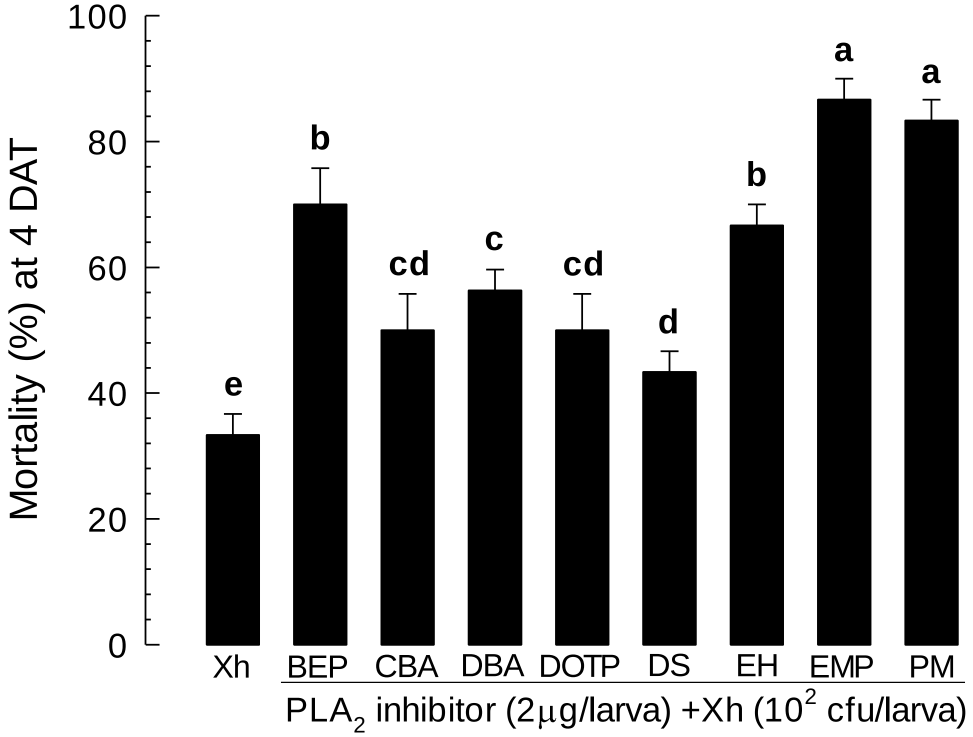
Mortality (%) at 4 DAT
100
80
60
40
20
0
e
b
cd
c
cd
d
b
a
a
Xh
BEP
CBA
DBA
DOTP
DS
EH
EMP
PM
PLA2 inhibitor (2µg/larva) +Xh (10^2 cfu/larva)

(B)

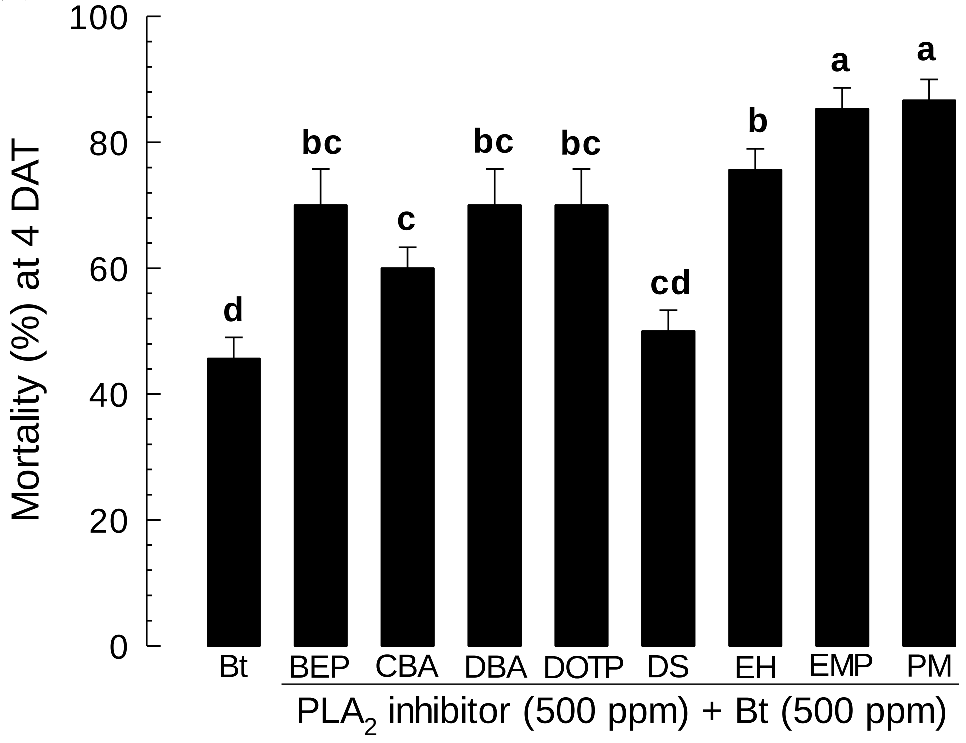
Mortality (%) at 4 DAT
100
80
60
40
20
0
d
bc
c
bc
bc
cd
b
a
a
Bt
BEP
CBA
DBA
DOTP
DS
EH
EMP
PM
PLA2 inhibitor (500 ppm) + Bt (500 ppm)

Fig. S6
